# Visceral Leishmaniasis Treatment Outcome and Associated Factors in Northern Ethiopia

**DOI:** 10.1155/2019/3513957

**Published:** 2019-08-21

**Authors:** Kidu Gidey, Desalegn Belay, Berhane Yohannes Hailu, Tesfaye Dessale Kassa, Yirga Legesse Niriayo

**Affiliations:** Department of Clinical Pharmacy, School of Pharmacy, College of Health Sciences, Mekelle University, Mekelle, Ethiopia

## Abstract

**Background:**

Visceral leishmaniasis (VL), one of the most neglected tropical diseases, is placing a huge burden on Ethiopia. Despite the introduction of antileishmanial drugs, treatment outcomes across regions are variable due to drug resistance and other factors. Thus, understanding of VL treatment outcomes and its contributing factors helps decisions on treatment. However, the magnitude and the risk factors of poor treatment outcome are not well studied in our setting. Therefore, our study was designed to assess treatment outcomes and associated factors in patients with VL.

**Materials and Methods:**

A cross-sectional study was conducted in VL patients admitted between June 2016 and April 2018 to Ayder Comprehensive Specialized Hospital, Tigray, Northern Ethiopia. Data was collected through chart review of patient records. Logistic regression analysis was used to identify factors associated with poor treatment outcome.

**Results:**

A total of 148 VL patients were included in the study. The mean age (SD) of the patients was 32.86 (11.9) years; most of them (94.6%) were male patients. The proportion of poor treatment outcome was 12.1%. Multivariable logistic regression analysis showed that long duration of illness (> four weeks) (adjusted odds ratio (AOR): 6.1 [95% confidence interval (CI); 1.3-28.6], p=0.02) and concomitant tuberculosis (TB) infection (AOR 4.6 [95% CI; 1.1-19.1], p=0.04) were the independent predictors of poor treatment outcome.

**Conclusions:**

Poor treatment outcome was observed in a considerable proportion of VL patients. Long duration of illness and coinfection with TB were associated with poor VL treatment outcome. Hence, early diagnosis and effective prompt treatment are important to improve treatment outcomes among VL patients. Special attention should also be given in the treatment of VL/TB coinfected patients in our setting.

## 1. Introduction

Visceral leishmaniasis (VL) is one of the most neglected tropical diseases caused by protozoa of the genus* Leishmania* [1]. The disease is transmitted to humans through the bite of infected female phlebotomine sandflies [2]. Some animal species, including domestic dogs, rodents, and carnivores, are the common host reservoir of VL in East Africa [3]. Among the species of* Leishmania*,* L. donovani* and* L. infantum* are known to give rise to the visceral form of the disease.* L. donovani *is commonly found in East Africa and the Indian subcontinent, while* L. infantum* occurs in Europe, North Africa, and Latin America [4]. VL is becoming a growing public health threat, imposing an estimated disease burden of more than 50,000 deaths each year around the world [5]. Over 90% of global cases of VL have been reported in six countries, including India, Bangladesh, Sudan, South Sudan, Ethiopia, and Brazil [6].

In Ethiopia, VL is caused by* L. donovani*, which is still endemic and prevalent [3]. Among the Sub-Saharan African countries, Ethiopia has recorded the highest number of VL cases next to Sudan. The annual incidence of VL in Ethiopia is estimated to be between 4,500 and 5,000 cases, with a population at risk of more than 3.2 million [7].

Visceral leishmaniasis is highly fatal if left untreated in over 95% of cases [8]. During the past decades, the introduction of antileishmanial drugs has resulted in a remarkable reduction of mortality [9]. However, many challenges remain to be overcome including the availability of only a few chemotherapeutic agents, stock-out of drugs, cost of treatment, adverse effects, and treatment failure [10, 11]. Poor treatment outcomes among patients with VL were reported in several studies worldwide [10, 12, 13]. Moreover, all drugs currently used for VL, except amphotericin B, are prone to the development of resistance [14]. Because of the paucity of drugs in the treatment of VL, it is very important to prevent the emergence of further drug-resistant strains [15].

In Ethiopia, the first-line drugs for the management of VL are a combination of antimonials with aminoglycosides and Liposomal Amphotericin B (LAMB) for special situations (for HIV-positive and severely ill immunocompetent VL patients) [16, 17]. Despite the introduction of these drugs, mortality is still high in Ethiopia. This is directly or indirectly related to poor treatment outcomes, which occur for a number of reasons [18]. The risk factors for poor treatment outcome and response for the different antileishmanial drug are highly variable in different populations. For example, the rate of resistance for sodium stibogluconate (SSG) is very high in the Indian population unlike the east African countries with low resistance [19–21]. Risk factors for poor treatment outcome are also variable and include age, duration of illness, malnutrition, presence of anemia, confections, ethnicity, and high baseline parasite load [9, 22–26].

Thus, determination of local data about treatment outcome and the risk factors of poor treatment outcome is very crucial for national control, prevention, and elimination of VL. A better understanding of treatment outcomes helps policymakers, clinicians, and funding organizations to develop strategies for improving outcomes. Studies that determine VL treatment outcome at some interval are vital to prevent the occurrence of drug resistance in our region even though resistance is not a common problem in East Africa at the present time. However, limited data are available in Ethiopia in general and there is no study on treatment outcome of VL patients in our setting. The present study aimed to assess the treatment outcome and associated factors among VL patients at Ayder Comprehensive Specialized Hospital (ACSH), northern Ethiopia.

## 2. Materials and Methods

### 2.1. Study Setting

The study was conducted at ACSH, Tigray region, Northern Ethiopia. ACSH is a teaching and referral hospital with 500 beds. The hospital serves for more than 9 million people in the catchment area. It provides both outpatient and in-patient services.

### 2.2. Study Design and Population

We conducted an institutional based cross-sectional study. The study was conducted using retrospectively collected data. All adult patients (age >18 years) with a well-established diagnosis of VL who were admitted to ACSH from June 01/2016 to April 30/2018 were included consecutively. Diagnosis of VL was performed according to Ethiopian Ministry of Health guidelines using a positive recombinant kinesin antigen (rK39) rapid diagnostic test (DiaMed-IT-Leish, DiaMed, Cressier, Switzerland) and the microscopic examination of aspirates from the spleen and/or bone marrow in a patient presented with sign and symptoms suggestive of VL [17]. Patients who were transferred out to other hospitals (5) and those patients having incomplete medical record (3) were excluded from the study.

### 2.3. Ethical Considerations

The ethical approval and clearance were obtained from Ethics Review Committee of the School of Pharmacy, College of Health Sciences, Mekelle University (reference number: CHS/158/pharm-10). Permission was obtained from the hospital's medical director to access the patient's medical records. The privacy of personal information was strictly conserved.

### 2.4. Definition of Terms and Outcome Measures

VL case definition is the following: “A person who presents with fever for more than two weeks and an enlarged spleen (splenomegaly) and/or enlarged lymph nodes (lymphadenopathy), or either loss of weight, anemia or leucopenia while living in a known VL endemic area or having travelled to an endemic area” [17]. Anemia was defined as hemoglobin (HGB) <13 g/dl in males and <12 g/dl in females, according to WHO criteria [27]. Leukopenia was defined as the number of white blood cells <4500/dL and thrombocytopenia was defined as the number of platelets <150 000/dL. Good outcome was defined as a cure at the end of treatment, improvement of signs and symptoms after completion of standard treatment (resolution of fever, increase in hemoglobin, weight gain, and regression of spleen size), and absence of parasites on the smears. Treatment failure was defined as the presence of clinical signs/symptoms, the presence of parasites in the smear, and/or positive test of cure cultures that persist after standard treatment is completed. Death was defined as mortality for any reason during treatment. Poor treatment outcome was defined as the experience of death or treatment failure.

### 2.5. Treatment and Follow-Up

The treatment in the present study was done using either the combination of SSG and paromomycin (PM) or LAMB. SSG (20mg/kg /day) and* PM* (15mg/kg /day) injections were given for 17 days. Weekly weight was measured and dosage adjustment was done accordingly. The dose of LAMB used was 5mg/kg/day over a period of 6 days. The combination of SSG-PM was used for majority of the patients except some patients with HIV and patients having contraindications like severe anemia. LAMB was used for all HIV patients and for patients with treatment failure from SSG and PM. Patients were followed until the end of treatment and were discharged or until death.

### 2.6. Data Collection

Demographic, clinical, laboratory, and treatment related factors were collected using a data extraction tool. These variables were collected from the patients' medical record by trained health professionals.

### 2.7. Statistical Methods

Data were entered into an Epi data management (version 4.2.0) and analyzed using the Statistical Package (IBM SPSS Statistics for Windows, Version 21.0. Armonk, NY: IBM Corp). Descriptive analysis was computed using mean and median for quantitative variables and frequency (percentage) for categorical variables. Multicollinearity test was performed to determine correlation among independent variables using variance inflation factor (VIF) and there was no collinearity (VIF<2 for all the variables). A univariate logistic regression analysis was performed to determine the association of each independent variable with the outcome. Subsequently, variables with a p-value<0.25 in the multivariable analysis were included in the multivariable logistic regression model to identify predictors of poor treatment outcome. A p-value of <0.05 (*α*=0.05) was used to declare a statistically significant association.

## 3. Results

### 3.1. Demographic and Clinical Characteristics of VL Patients

A total of 148 VL patients were included in this study. Of these, 94.6% were males. The mean (SD) age of the patients was 32.86 ± 11.9 years. Majorities (91.6%) were from rural areas. Fever, weight loss, and loss of appetite were present in all patients. The majority (76.4%) of the participants were newly diagnosed VL patients. The median duration of the illness was 28 days with interquartile range (IQR) from 14 to 56 days. All patients were rK39 positive and majorities (74%) of the patients were splenic aspirate positive. Majorities (95.9%) of the patients had anemia, leucopenia (93.9%), and thrombocytopenia (84.5%). TB coinfection was presented in 24.3% of the patients. Multiple coinfection with HIV-TB-VL coinfection was present in 10.1% of patients and malaria in 27% of patients (Table 1).

### 3.2. Treatments and Outcome of VL Patients

Majorities (71.6%) of the patients were treated using a combination of SSG & PM. A blood transfusion was administered to 24.3% of the patients. Anti-TB drugs were administered to 24.3% of the patients and 16.9% of the patients were on ART. Our finding revealed that majorities (87.9%) of the patients had good treatment outcome/cured and 12.1% had poor treatment outcome (6.7% of the patients had treatment failure and 5.4% died) (Table 2).

### 3.3. Factors Associated with Poor Treatment Outcome of VL

A univariate logistic regression analysis was performed to determine the association of each independent variable with the outcome (Table S1). Subsequently, variables with a p-value<0.25 in the univariate analysis were included in the multivariate logistic regression model to assess the predictive factors of poor treatment outcome.

The model contained seven independent variables and the full model containing all predictors was statistically significant (X^2^ = 28.96; df = 10; N= 148; p-value=0.001). The results of the multivariate logistic regression indicated that patients who were hospitalized lately after the clinical manifestation (long duration of illness > four weeks) were more likely to have poor treatment outcome (adjusted odds ratio (AOR):* 6.08,* 95% confidence interval (CI): 1.29-28.55) than patients who were diagnosed early. Similarly, patients with concomitant infection of TB were more likely to have poor treatment outcome (AOR= 4.55, 95% CI: 1.08-19.13) (Table 3).

## 4. Discussion

We found that 12.1% of patients had poor treatment outcomes, similar to the rate seen in East Africa [28]. However, this result was lower compared to previous findings by Lyons et al. (18.5%) in the same region in 2003 [29]. This may be due to differences in the characteristics of the subjects involved in the study and the type of treatment used. In the previous study, subjects with HIV coinfection were higher and may result in poorer treatment outcomes. In addition, in the present study, the combination of SSG and PM or LAMB was used in contrast to the previous study that used only SSG. The combination of SSG and PM is superior to SSG alone [28], and the better treatment outcome in the current study may be due to the use of this preferred regimen. Higher rates of poor treatment outcome have been also reported in other studies conducted in Northwest Ethiopia (23.7%) [9] and India (22.7%) [30]. However, since leishmaniasis is a treatable and curable disease, the proportion of poor treatment in our study is still high and was higher than in other studies conducted in East Africa (4.9%) [28] and Sudan (8%) [25]. These variabilities in the rate of poor treatment outcome may arise from the differences in setting, characteristics of participants, the type of treatment used, and study design.

The study also determined the predictive factors of poor VL treatment outcomes. Long duration of illness and coinfection with TB were associated with poor treatment outcome. We found that patients with long duration of illness (> four weeks) were six times more likely to have poor treatment outcome (AOR: 6.08 [95% CI: 1.29-28.55], p=0.02). Poor treatment outcome related to the delay in diagnosis was reported in other similar studies [25, 31]. The implication of this finding is the importance of early diagnosis, as the disease is completely curable with timely treatment [32]. Furthermore, transmission can be reduced through early case detection followed by complete treatment [10]. Thus, health care providers and public education are very crucial to reduce the time from symptom onset to diagnosis.

Patients with concomitant TB infection were more likely to have a poor outcome (AOR 4.55 [95% CI: 1.08-19.13], p=0.04). In a study conducted in Bangladesh, about 8% of the deceased patients had TB, indicating the existence of VL/TB coinfection [23]. A systematic review of the coinfection of TB and parasitic diseases revealed that there is an association between VL and TB in several endemic VL countries [33]. The presence of TB was also an independent predictor of poor treatment outcome among VL and HIV coinfected patients [34]. Therefore, evaluation of concomitant TB infection and appropriate treatment in patients with VL diagnosis is very crucial.

In our study, around 20% of the patients were coinfected with HIV. Despite the many studies which reported a significant association between HIV and VL [9, 29, 35], we did not find a significant association between poor treatment outcome and HIV coinfection. This could be as a result of the better care provided in HIV patients in recent times. Moreover, the self-care practice of HIV patients is becoming good and such patients might visit health institutions early.

The results of this study have important implications. The poor treatment outcomes seen in a significant proportion of patients require special attention. Policy makers and health care providers should be involved in improving treatment outcomes. Since this study shows that longer pretreatment duration is an important predictor, attention should be paid to early diagnosis of VL patients. Diagnosis can be made early through active case finding, which was an effective component for eliminating leishmaniasis in the Indian subcontinent [36]. Thus, policy makers should work at community level to prevent delay in diagnosis through active case search. As a priority, those patients with coinfections should be screened for sign and symptoms of VL. Health education should also be provided to improve the health seeking behaviors of patients.

### 4.1. Limitation of the Study

Finally, a number of important limitations need to be considered. The data were extracted retrospectively from the patients' medical chart and some important information was not consistently available (e.g., bone marrow aspiration result). The safety data for antileishmanial drugs were incomplete and were not presented in this study. The cross-sectional nature of our study may not provide adequate evidence of causality regarding the risk factor for the poor treatment outcome. We were also unable to assess the long-term treatment outcome (treatment outcome at 6 months), since most of the VL patients were from rural areas and did not have follow-up data after the initial discharge. Long-term outcomes of the disease in our setting remain to be investigated.

## 5. Conclusion 

Poor treatment outcome was observed in a considerable proportion of VL patients. Long duration of illness and coinfection with TB were associated with poor VL treatment outcome. Hence, early diagnosis and effective prompt treatment are important to improve treatment outcomes among VL patients. Special attention should also be given in the treatment of VL/TB coinfected patients in our setting.

## Figures and Tables

**Table 1:** Demographic and disease related characteristics of VL patients in ACSH, Tigray Region, Northern Ethiopia, June 2016–April 2018.

Variable	Category	Frequency (%)
Gender	Female	8 (5.4)
	Male	140 (94.6)
Age in years	18-35	94 (63.5)
	36-50	42 (28.4)
	>50	12 (8.1)
	Mean ± SD	*32* ± 11.939
Residence	Rural	136 (91.9)
	Urban	12 (8.1)
Treatment History	New case	113 (76.4)
	Previously treated	35 (23.6)
Duration prior to diagnosis (weeks)	< 2	42 (28.4)
	2-4	52 (35.1)
	> 4	54 (36.5)
Presence of signs and symptoms		
Fever	Yes	100 (100)
Body weakness	Yes	124 (83.8)
	No	24 (16.2)
Epistaxis	Yes	23 (15.5)
	No	125 (84.5)
Weight loss	Yes	148 (100)
Loss of appetite	Yes	148 (100)
Diarrhea	Yes	15 (10.1)
	No	132 (89.2)
Splenomegaly	Yes	145 (98)
Lymphadenopathy	No	03 (2.0)
	Yes	06 (4.1)
Anemia	Yes	142 (95.9)
	No	06 (4.1)
Leucopenia	Yes	139 (93.9)
	No	09 (6.1)
Thrombocytopenia	Yes	125 (84.5)
	No	23 (15.5)
Method of diagnosis		
** **Serologic (rk39 dipstick)	Positive	148 (100)
** **splenic aspirate result	Positive	110 (74.3)
	Negative	01 (0.7)
	Not done	37 (25)
Concomitant infection		
** **TB	Yes	36 (24.3)
	No	112 (75.7)
** **HIV Status	Positive	31 (20.9)
	Negative	117 (79.1)
** **HIV and TB coinfection	Yes	15 (10.1)
	No	133 (89.9)
** **Malaria	Yes	40 (27)
	No	108 (73)

**Table 2:** Medication related characteristics of VL patients at ACSH, Tigray Region, Northern Ethiopia, June 2016–April 2018.

Characteristics	Category	Frequency (%)
VL Treatment	SSG + PM	106 (71.6)
	LAMB	42 (28.4)
Concomitant treatment	Blood transfusion	36 (24.3)
	Antibiotics	15 (10.3)
	Anti-TB	36 (24.3)
	ART	25 (16.9)
Treatment outcome	Cured	130(87.9)
	Treatment failure	10 (6.7)
	Death	08 (5.4)

ART: antiretroviral therapy, LAMB: liposomal amphotericin B, PM: paromomycin, and SSG: sodium stibogluconate.

**Table 3:** Multivariable logistic regression analysis of predictors of poor treatment outcome among VL patients at ACSH, Tigray Region, Northern Ethiopia, June 2016–April 2018.

Variables	Category	Treatment outcome		COR (95% CI)	P- value	AOR (95% CI)	P -Value
		Good, n (%)	Poor, n (%)				
Age in years	18-35	84(64.6)	10(55.6)	1		1	
	36-50	37(28.5)	5(27.8)	1.14(0.36, 3.55)	0.83	0.83(0.22, 3.20)	0.78
	>50	9(6.9)	3(16.7)	2.8(0.65,12.08)	0.17	4.80(0.87, 26.46)	0.07
Duration prior to diagnosis (weeks)	<2	39(30)	3(16.7)	1		1	
	2-4	50(38.5)	2(11.1)	0.52(0.83, 3.23)	0.48	0.56(0.07, 4.25)	0.58
	>4	41(31.5)	13(72.2)	*4.12(1.09, 15.58)*	*0.04*	*6.08(1.29, 28.55)*	*0.02*
Body weakness	No	19(14.6)	5(27.8)	1		1	
	Yes	111(85.4)	13(72.2)	0.44(0.14, 1.39)	0.164	0.69(0.18, 2.68)	0.59
Epistaxis	No	112(86.2)	14(77.8)	1		1	
	Yes	18(13.8)	4(22.2)	2.39(0.76, 7.52)	0.135	3.22(0.73, 14. 31)	0.12
TB	No	104(80)	8(44.4)	1		1	
	Yes	26(20)	10(55.6)	*5 (1.80, 13.92)*	*0.002*	*4.55 (1.08, 19.13)*	*0.04*
HIV Status	No	105(80.8)	12(66.7)	1		1	
	Yes	25(19.2)	6(33.3)	2.10(0.72, 6.12)	0.17	1.29(0.12, 14.56)	0.83
Malaria	No	97(74.6)	11(61.1)	1		1	
	Yes	33(25.4)	7(38.9)	1.87(0.67, 5.22)	0.23	2.30(0.63, 8.43)	0.21
TB/HIV coinfection	No	120 (92.3)	13 (72.2)	1		1	
	Yes	10 (7.7)	5 (27.8)	*4.62(1.37, 15.58)*	*0.014*	2.68(0.14, 50.64)	0.51

AOR: adjusted odds ratio, CI: confidence interval, and HIV: human immune deficiency virus.

## Data Availability

The dataset of this study is available from the corresponding author upon request.

## Supplementary Materials

Supplementary Materials Table S1: univariable logistic regression analysis of predictors of poor treatment outcome among VL patients at ACSH, Tigray Region, Northern Ethiopia, June 2016–April 2018.
