# Supplementary material for: Visceral Leishmaniasis Treatment Outcome and Associated Factors in Northern Ethiopia
Source: Biomed Res Int. 2019 Aug 21;2019:3513957. doi: 10.1155/2019/3513957 (PMC6719273; doi:10.1155/2019/3513957)
Supplement: Supplementary Materials — Table S1: univariable logistic regression analysis of predictors of poor treatment outcome among VL patients at ACSH, Tigray Region, Northern Ethiopia, June 2016–April 2018. [file 3513957.f1.docx]

Table S1. Univariable logistic regression analysis of predictors of poor treatment outcome among VL patients at ACSH, Tigray Region, Northern Ethiopia, June 2016 – April 2018 (n=148)

| Variables | Category | Treatment outcome | | COR (95% CI) | P. value |
| --- | --- | --- | --- | --- | --- |
|  |  | Good, n (%) | Poor, n (%) |  |  |
| Gender | Female | 7(5.4) | 1(5.6) | 1 |  |
|  | Male | 123(94.6) | 17(94.4) | 0.97(0.11, 8.3) | 0.98 |
| Age in years  Mean (SD) Age = 32.66 (11.94) | 18-35 | 84(64.6) | 10(55.6) | 1 |  |
|  | 36-50 | 37(28.5) | 5(27.8) | 1.14(0.36, 3.55) | 0.83 |
|  | >50 | 9(6.9) | 3(16.7) | 2.8(0.65,12.08) | 0.17 |
| Residence | Rural | 118(90.8) | 16(88.9) | 1 |  |
|  | Urban | 12(9.2) | 2(11.1) | 0.81(0.17, 3.97) | 0.80 |
| Duration prior to diagnosis (weeks) | <2 | 39(30) | 3(16.7) | 1 |  |
|  | 2-4 | 50(38.5) | 2(11.1) | 0.52(0.83, 3.23) | 0.48 |
|  | >4 | 41(31.5) | 13(72.2) | 4.12(1.09, 15.58) | 0.037 |
| Patient treatment history | New case | 99(76.2) | 14(77.8) | 1 |  |
|  | Relapse | 31(23.8) | 4(22.2) | 0.912(0.28, 2.97) | 0.879 |
| Body weakness | No | 19(14.6) | 5(27.8) | 1 |  |
|  | Yes | 111(85.4) | 13(72.2) | 0.44(0.14, 1.39) | 0.164 |
| Epistaxis | No | 112(86.2) | 14(77.8) | 1 |  |
|  | Yes | 18(13.8) | 4(22.2) | 2.39(0.76, 7.52) | 0.135 |
| Anemia | No | 5(3.8) | 1(5.6) | 1 |  |
|  | Yes | 125(96.2) | 17(94.4) | 1.47(0.16, 13.35) | 0.73 |
| Leucopenia | No | 8(6.2) | 1(94.3) | 1 |  |
|  | Yes | 122(93.8) | 17(94.4) | 1.12(0.13, 9.47) | 0.921 |
| Thrombocytopenia | No | 23(17.7) | 2(11.1) | 1 |  |
|  | Yes | 107(82.3) | 16(88.9) | 1.72(0.37, 8.01) | 0.49 |
| VL treatment | SSG-PM | 94(72.3) | 12(66.7) | 1 |  |
|  | L-AMB | 36(27.7) | 6(33.3) | 1.31(0.46, 3.74) | 0.62 |
| Tuberculosis | No | 104(80) | 8(44.4) | 1 |  |
|  | Yes | 26(20) | 10(55.6) | 5 (1.80, 13.92) | 0.002 |
| HIV Status | No | 105(80.8) | 12(66.7) | 1 |  |
|  | Yes | 25(19.2) | 6(33.3) | 2.10(0.72, 6.12) | 0.17 |
| Malaria | No | 97(74.6) | 11(61.1) | 1 |  |
|  | Yes | 33(25.4) | 7(38.9) | 1.87(0.67, 5.22) | 0.23 |
| TB/HIV coinfection | No | 120 (92.3) | 13 (72.2) | 1 |  |
|  | Yes | 10 (7.7) | 5 (27.8) | 4.62(1.37, 15.58) | 0.014 |

^AOR: adjusted odds ratio, CI: confidence interval, COR: crude odds ratio, HIV: human immune deficiency virus, L-AMB (Liposomal amphotericin, SSG-PM: sodium stibogluconate and paromomycin,^
